# Multiscale Analysis of Electrocatalytic Particle Activities: Linking Nanoscale Measurements and Ensemble Behavior

**DOI:** 10.1021/acsnano.3c06335

**Published:** 2023-10-26

**Authors:** Minkyung Kang, Cameron L. Bentley, J. Tyler Mefford, William C. Chueh, Patrick R. Unwin

**Affiliations:** † School of Chemistry, The University of Sydney, Camperdown 2006 NSW, Australia; ‡ Department of Chemistry, The University of Warwick, Coventry CV4 7AL, U.K.; § School of Chemistry, Monash University, Clayton 3800 VIC, Australia; ∥ Department of Materials Science and Engineering, Stanford University, Stanford, California 94305, United States

**Keywords:** electrocatalysis, scanning electrochemical cell microscopy, oxygen evolution reaction, single-entity electrochemistry, multiscale electrochemical analysis

## Abstract

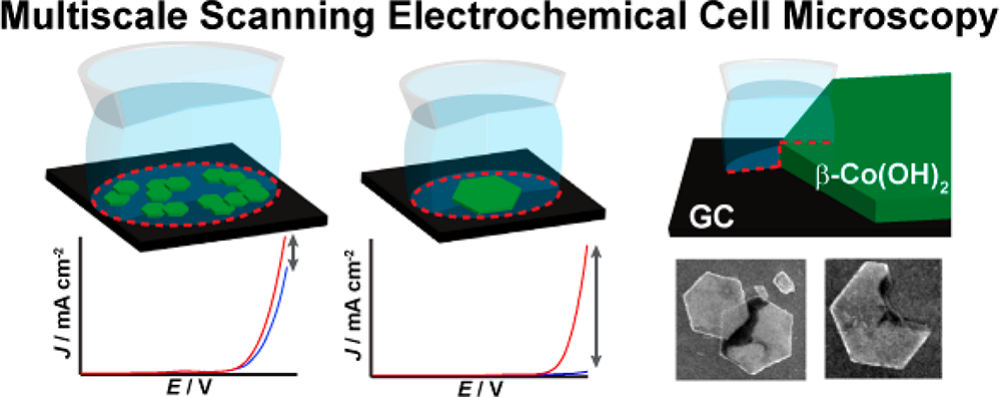

Nanostructured electrocatalysts exhibit variations in electrochemical properties across different length scales, and the intrinsic catalytic characteristics measured at the nanoscale often differ from those at the macro-level due to complexity in electrode structure and/or composition. This aspect of electrocatalysis is addressed herein, where the oxygen evolution reaction (OER) activity of β-Co(OH)_2_ platelet particles of well-defined structure is investigated in alkaline media using multiscale scanning electrochemical cell microscopy (SECCM). Microscale SECCM probes of ∼50 μm diameter provide voltammograms from *small particle ensembles* (ca. 40–250 particles) and reveal increasing dispersion in the OER rates for samples of the same size as the particle population within the sample decreases. This suggests the underlying significance of heterogeneous activity at the single-particle level that is confirmed through *single-particle* measurements with SECCM probes of ∼5 μm diameter. These measurements of multiple individual particles directly reveal significant variability in the OER activity at the single-particle level that do not simply correlate with the particle size, basal plane roughness, or exposed edge plane area. In combination, these measurements demarcate a transition from an “individual particle” to an “ensemble average” response at a population size of ca. 130 particles, above which the OER current density closely reflects that measured in bulk at conventional macroscopic particle-modified electrodes. Nanoscale SECCM probes (ca. 120 and 440 nm in diameter) enable measurements at the *subparticle level*, revealing that there is selective OER activity at the edges of particles and highlighting the importance of the three-phase boundary where the catalyst, electrolyte, and supporting carbon electrode meet, for efficient electrocatalysis. Furthermore, subparticle measurements unveil heterogeneity in the OER activity among particles that appear superficially similar, attributable to differences in defect density within the individual particles, as well as to variations in electrical and physical contact with the support material. Overall this study provides a roadmap for the multiscale analysis of nanostructured electrocatalysts, directly demonstrating the importance of multilength scale factors, including particle structure, particle–support interaction, presence of defects, etc., in governing the electrochemical activities of β-Co(OH)_2_ platelet particles and ultimately guiding the rational design and optimization of these materials for alkaline water electrolysis.

Advances in nanoscience have seen the widespread adoption of nanostructured electrodes in all areas of modern electrochemical science, including environmental/biological sensing, electrocatalysis, and energy storage.^1−3^ Yet, despite the evident microscopic complexity, i.e., structural heterogeneity, of nanostructured electrodes, routine electrochemical characterization is still almost exclusively carried with classical macroscopic “bulk” techniques utilizing electrodes with geometric surface areas >0.01 cm^2^ such as rotating disk/ring electrochemistry, coin/pouch cell two-electrode measurements, membrane electrode assemblies, etc. Bulk electrochemistry provides the *ensemble-averaged* response of an electrode, which gives ready access to benchmarking metrics that are important for practical applications (e.g., specific activity, energy density, etc.). However, from bulk electrochemistry alone, it is difficult to decipher how the *intrinsic properties* of a nanomaterial (e.g., surface structure/composition) give rise to a particular macroscopic function (e.g., electrochemical activity, stability, selectivity, etc.).^4,5^ For this reason, there is a great need for different approaches that can effectively resolve *structure*–*function* at the nanoscale,^2^ which will not only enable optimization of existing electrochemical technologies that utilize nanostructured electrodes (e.g., batteries, fuel cells, supercapacitors, etc.) but also facilitate the rational design and development of advanced materials with enhanced function.

S*ingle-entity electrochemistry* (SEE)^6,7^ is a rapidly evolving field, in which electrochemical techniques are used to individually interrogate the *simple units* (e.g., a single step edge, particle, grain, grain boundary, etc.) that make up complex systems (e.g., nanostructured electrodes). Understanding the electrochemical properties of a single entity reveals its individual contribution to the ensemble average (i.e., macroscale or bulk electrochemical response), providing a *bottom-up* perspective of electrode structure–function. For particles, previous SEE studies have revealed unique electrochemical activities among ensembles of superficially similar particles,^8−13^ dynamic interactions between individual nanoparticles and the underlying support during electrocatalytic turnover,^14,15^ and heterogeneous particle–support contacts that limit battery charge/discharge capability.^16^*Multiscale electrochemistry* seeks to bridge the gap between the microscopic (single-entity) and macroscopic (ensemble) worlds to provide a holistic view of complex electrodes/electromaterials across length and time scales.

Among tools for SEE, scanning electrochemical cell microscopy (SECCM) is proving to be a particularly powerful and versatile technique that enables correlative structure–function studies in (electro)materials science.^6,17^ In SECCM, the meniscus cell formed at the end of a fluidic scanning probe (composed of a glass micropipet or nanopipet) is brought into contact with a target entity (i.e., an area of an electrode surface) to perform local electrochemistry with high spatiotemporal resolution. Employed in tandem with complementary, colocated high-resolution spectroscopy/microscopy in a *correlative multimicroscopy approach*, SECCM has previously been used to probe the activity of single step edges (e.g., transition-metal dichalcogenides^18−20^ and sp^2^ carbon^21−23^), nanoparticles (e.g., metal^8,9^ and metal oxides^11,12,24^), inclusions,^25,26^ grains^27−32^ and grain boundaries,^26,33^ etc. Two very recent SECCM studies on complex electrode materials demonstrate single-entity behavior that would not be readily predicted from bulk electrochemistry alone: (1) individual LiMn_2_O_4_ particles exhibit facile Li^+^ (de)intercalation at rates that are orders of magnitude higher than macroscopic composite electrodes of the same material^11^ and (2) individual conductive domains of poly(3-hexylthiophene) (P3HT) retain facile electron-transfer rate capability when blended with nonconductive poly(methyl methacrylate) (PMMA), despite apparently ultrasluggish electron transfer at the macro-scale.^4^ These studies highlight the need for techniques/methodology that can bridge the gap between the single-entity and macroscopic worlds,^34^ which is readily achievable in SECCM on the same platform through the use of a set of probes of graded dimensions.^35,36^

Herein we use multiscale SECCM to study the oxygen evolution reaction (OER) activity of β-Co(OH)_2_ platelet particles (referred to as *particles* herein for brevity), specially engineered to possess well-defined crystallographic facets at the terrace (basal plane) and step (edge plane) terminations.^37,38^ Transition metal oxides, such as cobalt (oxy)hydroxides, are a promising class of OER electrocatalyst that are known to undergo complex, voltage-dependent structural transitions through ion-coupled redox reactions (e.g., proton (de)intercalation) in aqueous alkaline media.^3,39^ There is still much debate on the active structure of cobalt (oxy)hydroxides under electrocatalytic turnover, but recent studies on β-Co(OH)_2_ particles (identical to those used herein) suggest that the OER occurs exclusively on the {112̅0} and {101̅0} edge plane facets, while the {0001} basal plane facet is inactive, directly observed from a range of *operando* nanoscale electrochemical techniques^37^ and supported by macroscopic electrochemistry and computational simulations.^38,40^

Bringing to bear the full capability of multiscale, multiscanning mode SECCM, in tandem with atomic force microscopy (AFM), scanning electron microscopy (SEM), and transmission electron microscopy (TEM), we study OER catalysis at well-defined β-Co(OH)_2_ particles (supported on glassy carbon, GC) at the subparticle, single-particle, and ensemble (ca. from 2 to 100s of particles) levels. With this approach, we are able to establish direct correlations between the structure and activity of nanomaterials while also developing a connection between nanoscale properties and the overall bulk activity. The experiments are designed to directly address three fundamental research questions. (1) Does ensemble activity scale linearly with the coverage of particles on the GC support? (2) Do similar individual particles possess similar activity? (3) How do nanoscale structural features influence activity at the subparticle level, including the roles of crystallographic defects and the nature of the particle–support interaction? This study serves as a roadmap for the multiscale electrochemical analysis of nanomaterials in electrocatalysis and beyond, which will ultimately facilitate the rational design and optimization of highly active nanostructured electrodes.

## Results and Discussion

### Multiscale SECCM: Practical Considerations and Setup

**Figure 1:**
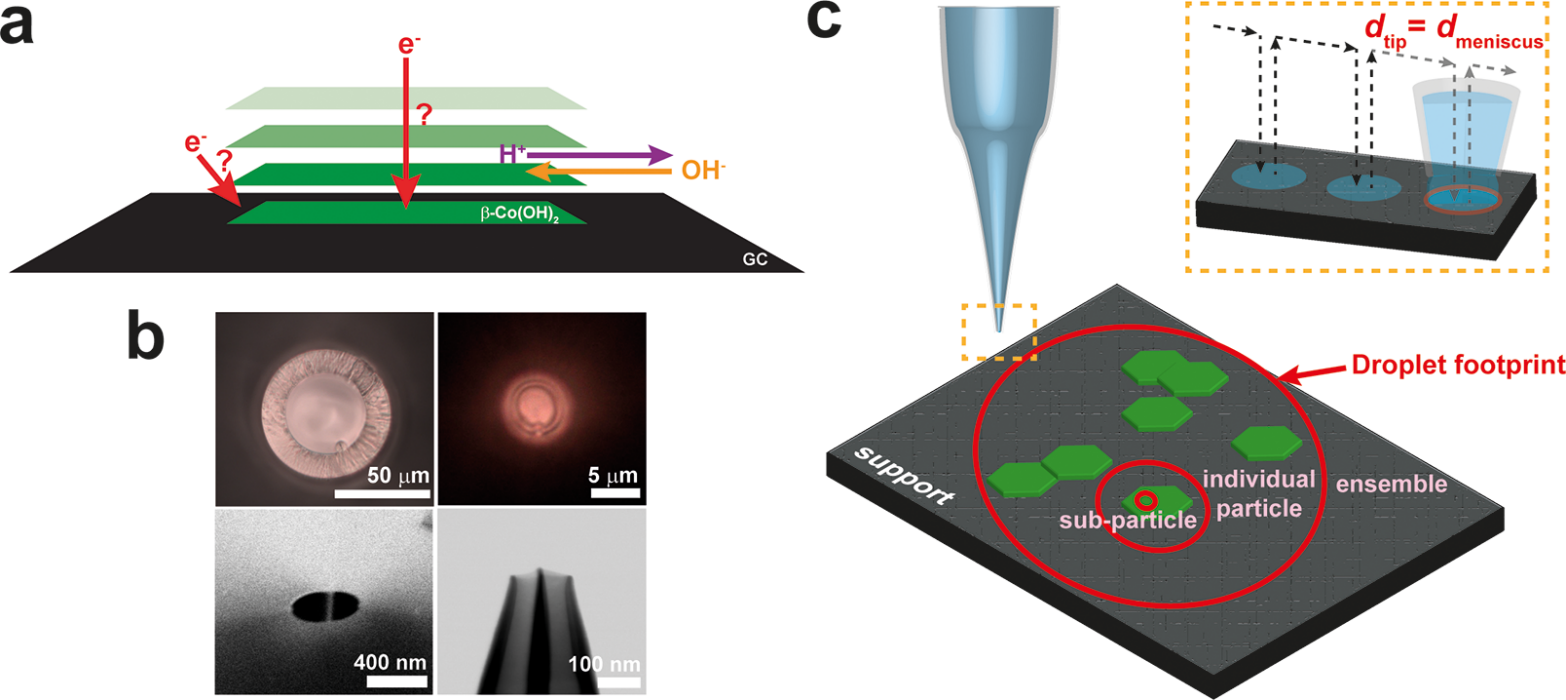
(a) Schematic illustrating the possible electron-transfer and ion transport pathways during OER catalysis at the β-Co(OH)_2_ particle supported on GC. (b) Two optical (top), one scanning electron microscopy (SEM) (bottom left), and one scanning transmission electron microscopy (STEM) (bottom right) images of SECCM probes that were used for SECCM. (c) Multiscale SECCM and how the size of the probe determines the length scale of the measurement: ensemble, individual particle, or subparticle. The diameter of the tip (*d*_tip_) corresponds approximately to the diameter of the meniscus (*d*_meniscus_) in the hopping mode (inset).

Multiscale SECCM can be readily achieved by using a series of different tip orifice sizes, as the dimensions of the electrochemical cell are determined by the size of the electrolyte meniscus formed naturally at the end of the probe (Figure 1). Considering the dimensions of the β-Co(OH)_2_ particles, i.e., an average edge length of approximately 1.5 μm and an average thickness of approximately 75 nm,^38^ probes of various sizes ranging from 55 μm to 120 nm in diameter, *d*_tip_ (Figure 1), were used for analysis at different length scales. For the measurement of both particle ensembles and individual particles, a single-channel probe was employed, using *i*_surf_ positional feedback. This mode was appropriate at these length scales because the dimensions of the meniscus cell guaranteed that it would entirely encapsulate the particle(s) and make electrolyte contact with the underlying GC support. A probe with *d*_tip_ = 55 μm typically encapsulated up to hundreds of particles, while a probe with *d*_tip_ = 6 μm ensured full coverage of a single particle (i.e., *single-entity* level measurement).

By contrast, analyzing the β-Co(OH)_2_ particles at the *subentity level* required the use of smaller probes (<500 nm, herein) in the dual-channel format due to the low intrinsic electronic conductivity of the {0001} facet of β-Co(OH)_2_.^37^ The use of a dual-channel probe provided supplementary positional feedback from the ionic current (i.e., *i*_DC_ or *i*_AC_). Probes with *d*_tip_ sizes of 440 and 120 nm were both used to investigate the role of the edge plane of the particle and the nature of the physical (electrical) contact between the particle and GC support in the observed OER activity. It should be noted that a thin layer of oil, a nonpolar and low dielectric medium,^41^ was applied to the surface to prevent micro-/nanoscale droplet leakage during scanning.^28,42,43^ Without the oil layer there would be excessive wetting of the surface by the SECCM meniscus under basic conditions.^44^ The presence of the oil layer has no impact on the physical contact between the particle and the GC support nor does it cause chemical contamination of the active site of the OER on the particle (see Figures S1 and S2 in the Supporting Information).

### Ensemble-to-Ensemble Variations in OER Activity at the Microscale

**Figure 2:**
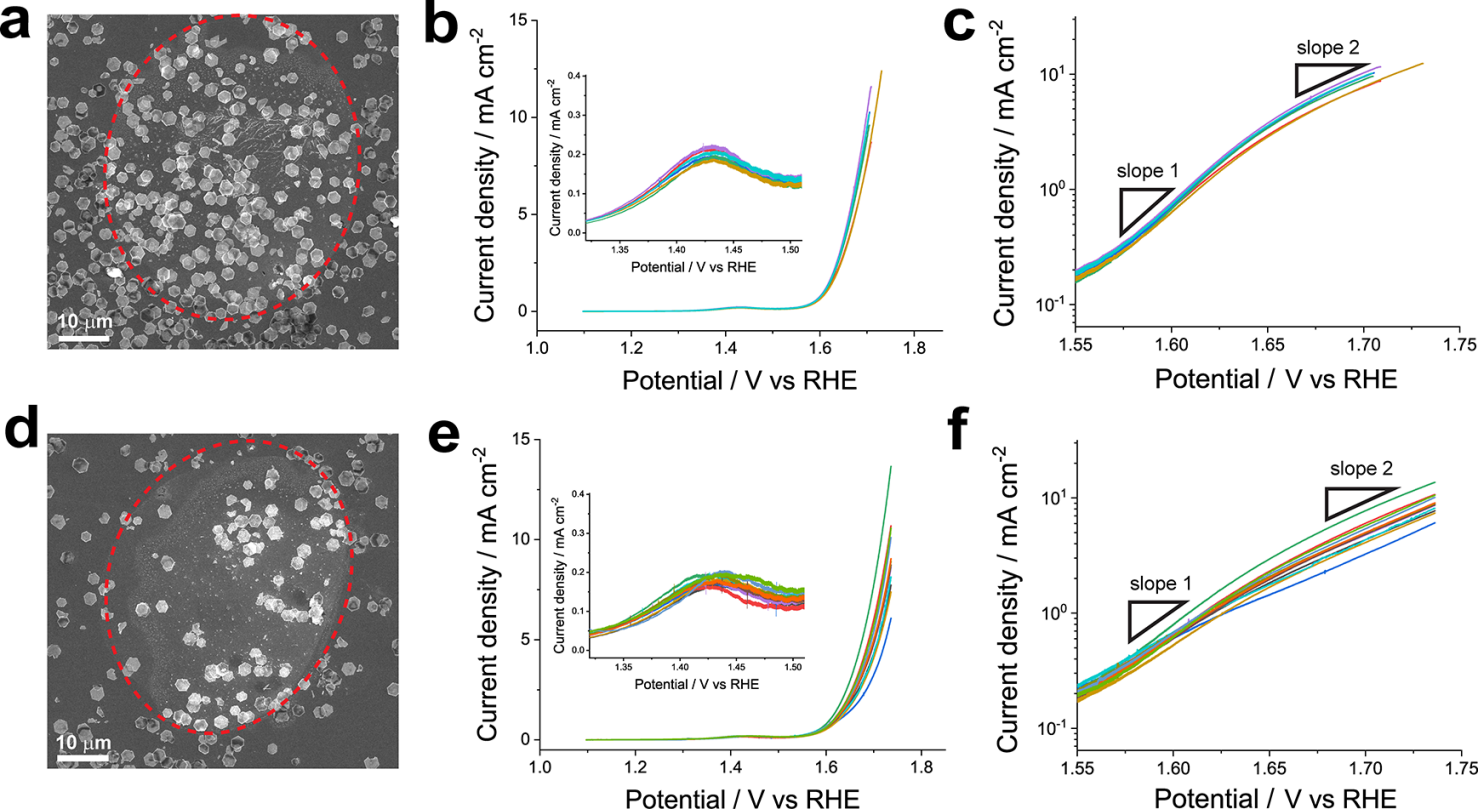
Comparisons of the OER activities of HD and LD β-Co(OH)_2_ particle ensembles, supported on GC. Representative SEM images for (a) HD and (d) LD particle ensembles with an outline (dotted red), indicating the perimeter of the meniscus cell during SECCM measurements. LSVs (υ = 10 mV s^–1^) of individual (b) HD (*N* = 6) and (e) LD (*N* = 12) particle ensembles and their corresponding (c, f, respectively) Tafel plots. The SECCM experiments were carried out using a single-channel micropipet probe with *d*_tip_ = 55 μm filled with 0.1 M KOH.

We begin with our studies using the largest scale probes. As alluded to above, a single channel probe with *d*_tip_ = 55 μm can encapsulate anywhere from 30 to over 100 particles in the meniscus cell during a single SECCM meniscus landing. Thus, each measurement performed at this scale represents the OER activity of a small particle ensemble (Figure 2), conceptually similar to a conventional “bulk” ensemble measurement of this material. Voltammetric hopping mode SECCM was deployed^30,45^ using a hopping distance of 100 μm; LSVs were acquired from 28 unique particle ensembles. The coverage of particles on the GC surface (θ) varied from 0.09 to 0.5 within the confined area. Based on the mean particle surface coverage of 0.26, the LSVs were categorized into high-density (HD) and low-density (LD) particle ensembles, corresponding to coverages of over 0.37 and below 0.22 (Figure 2a,d), respectively. Note that the current was normalized to current density using the projected particle ensemble area, which was obtained from the processed SEM images of individual particle ensembles (Figure S3 in the Supporting Information).

All particle ensembles exhibited LSVs (Figure 2b,e) that can be considered typical for the OER on β-Co(OH)_2_ particles, including an oxidation peak at 1.43 V vs RHE, corresponding to Co^2+/2.5+^ conversion, followed by the OER, which coincides with the further oxidation of Co (i.e., Co^2.5+/3+^) as the applied potential increased beyond 1.58 V vs RHE.^37^ Note that a generally linear relationship was observed between particle coverage (θ = 0.05–0.5; *N* = 28) and the OER activity, as indicated by the current measured at 1.71 V vs RHE (Figure S4 in the Supporting Information). However, there are distinct interensemble differences observed under HD versus LD conditions, which warrant further discussion.

The LSVs and corresponding Tafel plots obtained from six individual HD particle ensembles (θ = 0.37–0.50; *N* = 6) exhibited a high degree of similarity when normalized to current density, indicating a linear scaling between the particle coverage and the OER catalytic current. In other words, under HD conditions, the particles exhibited nearly identical OER activities from ensemble to ensemble (Figure 2b,c, *N* = 6). In all cases, the Tafel slope changes from 67 ± 3 mV dec^–1^ to 135 ± 3 mV dec^–1^ as more positive potentials are applied to the particles, reflecting typical variations in the active site density during the OER with increasing overpotentials.^37,46−48^ The results from LSVs and Tafel slope analysis on HD particle ensembles, in particular, are in good agreement with the macroscopic measurements using the same material.^37,38,49^ By contrast, the LD particle ensembles (Figure 2e,f, θ = 0.09–0.21; *N* = 12) exhibited more pronounced variations in the OER activity among individual ensembles/groups. These variations in LD particle ensembles were particularly evident in the potential-dependent Tafel behavior (see Table S1 in the Supporting Information). Notably, the standard deviation in the average Tafel slope, including both slope 1 at 1.58 V and slope 2 at 1.70 V, increased by 3-fold and 2-fold, respectively, under LD conditions compared to HD. To investigate the underlying cause of this apparent variability in OER activity/mechanism among LD particle ensembles, we investigated the particle-to-particle variation in activity using smaller SECCM probes, below.

### Particle-to-Particle Variations in Activity at the Microscale

An initial attempt was made to establish a link between the size and structure of individual β-Co(OH)_2_ particles and their unique OER activities through identical-location surface characterization alongside SECCM at the *single-particle level*. As noted above, the OER activity of β-Co(OH)_2_ particles is highly structure-sensitive, with the edge plane of the particles being particularly active.^37,38,40^ Thus, at the outset, it was postulated that the apparent OER activity of β-Co(OH)_2_ particles and particle ensembles should scale with the exposed edge plane area, possibly explaining the LD ensemble variation observed above.

To test this, an SECCM probe with a *d*_tip_ size of 6 μm was prepared, capable of encapsulating between 1 and 4 particles in a single measurement. Subsequently, voltammetric hopping mode SECCM (hopping distance of 10 μm) was performed on 22 individual particles (see Figure S5a in the Supporting Information). The OER activity varied substantially from particle to particle, with the average LSV (i.e., from all 22 particles) being consistent with that of the HD particle ensembles, above. For instance, the current density measured at 1.75 V vs RHE ranged from as low as 1.7 mA cm^–2^ to as high as 46.2 mA cm^–2^, depending on the particle. The locations of individual particles were identified in SEM, and the SEM images of the particles were categorized into three groups based on their OER activities: high, near-average, and low. Interestingly, there was no apparent correlation between the superficial shape and size of the individual particles and the degree of the OER activity (see Figure S5 in the Supporting Information).

**Figure 3:**
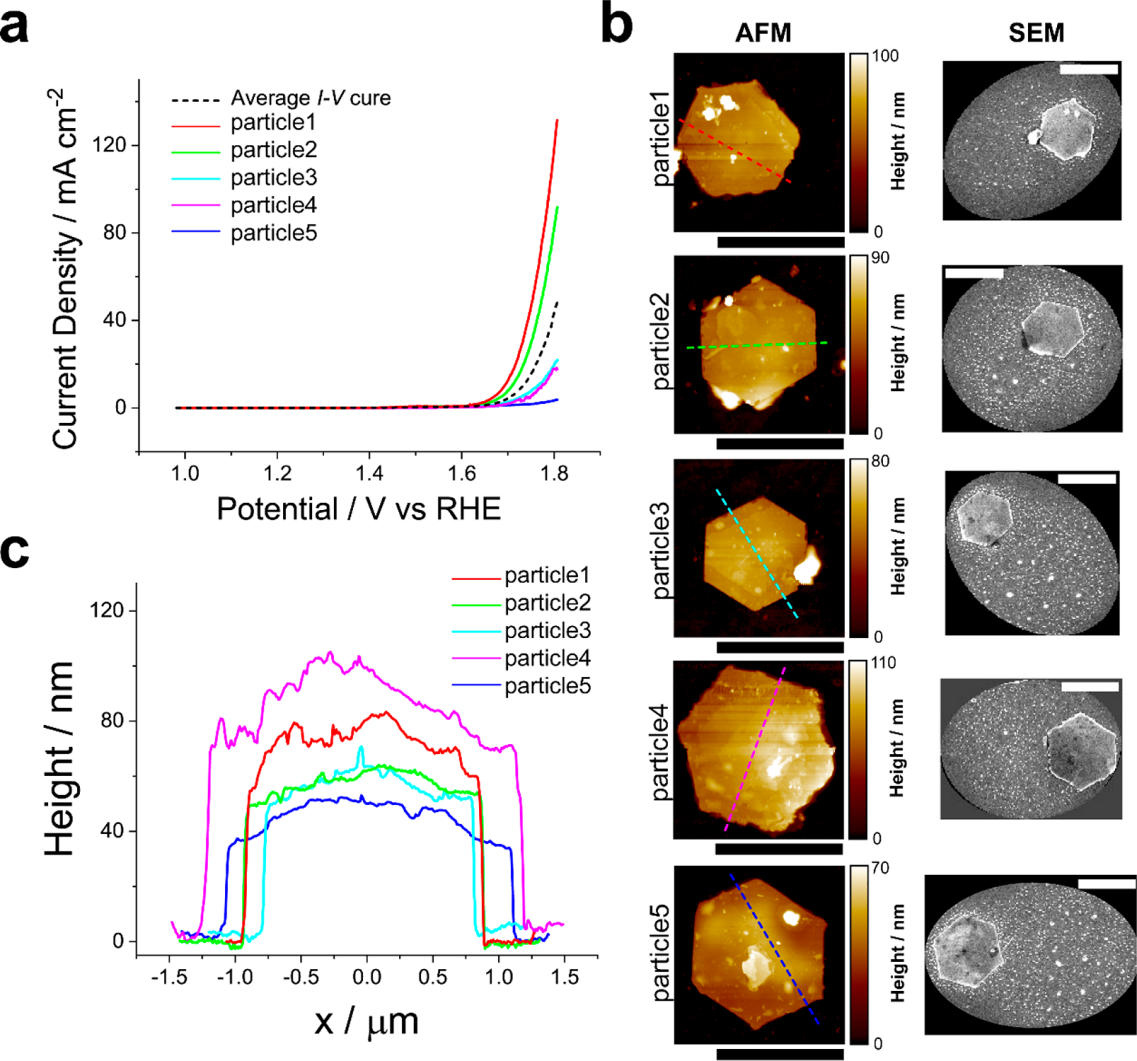
(a) LSVs (υ = 100 mV s^–1^) of five individual particles (solid-colored traces; labeled particle1–particle5) and the mean response from nine particles (dashed black trace). (b) Colocated AFM and SEM images of particle1–particle5. (c) AFM line profiles of height obtained across particle1–particle5, indicated by the dashed colored lines in (b). All scale bars are 2 μm. The SECCM experiments were carried out using a single-channel micropipet probe with *d*_tip_ = 6 μm filled with 0.1 M KOH.

Five particles were chosen for further analysis with *correlative multimicroscopy*,^17^ which are labeled as particle1 to particle5 (Figure 3a). These particles were chosen to cover the full range of activities (high, near-average, and low) among the population (*N* = 22) and possess OER activities that decrease in the order particle1 > particle2 > particle3 > particle4 > particle5. Identical-location SEM and AFM analyses (Figure 3b,c) were performed on particle1 to particle5 to correlate the OER activity of individual particles with the overall particle size, basal plane roughness, and/or estimated area of exposed edge plane (i.e., structure and height of step edges).

SEM imaging (Figure 3b) revealed no significant differences among the five particles, although the particle sizes varied slightly, in the order of particle4 > particle5 > particle1 ≈ particle2 > particle3. No major cracks or defects were observed on any of the particles. For a more detailed surface analysis, the average roughness of the basal planes of the particles (taken as a proxy for density of defects) was estimated from AFM imaging, considering that structural defects on the basal plane may contribute to the OER activity (*vide infra*). The estimated roughness ranged from 4 to 13 nm, with the order of particle4 > particle1 > particle5 > particle2 > particle3. Next, considering the significant role of the edge plane in the OER activity of β-Co(OH)_2_ particles,^37,40^ the edge plane surface area of the five particles was estimated from height profiles on particles from the AFM images. Despite the average step plane height being 75 nm,^38^ the height of individual particles varied from 38 to 77 nm (Figure 3c), decreasing in the order of particle4 > particle1 > particle2 > particle5 > particle3.

Evidently, none of the trends, in particle size, basal plane roughness, or edge plane area, in isolation, correspond to the observed order of OER activities on the individual particles. A clear example of this is demonstrated by particle3 and particle4. Despite particle4 possessing size, roughness, and edge plane area values that are >100% larger than those of particle3, both particles exhibit comparable activity in the measured LSV curves. In fact, particle3 has slightly higher activity (current) than particle4. This observation firmly emphasizes that particle size and apparent structural variations alone cannot explain the disparities observed in OER activities among individual particles.

The variations in the OER activity observed at the individual particle level mirror those in the LD particle ensembles, albeit with more pronounced differences between “low” and “high” (e.g., ca. 30-fold difference in Figure 3a, compared to ca. 3-fold difference in Figure 2e). The reason for this is explored below. Identical-location SEM and AFM analysis did not reveal a direct correlation between the OER activity and the size or structure of the particles, suggesting that other factors may be responsible. One possible explanation is that it is due to a combination of the low intrinsic electrical conductivity of the β-Co(OH)_2_ material^50−52^ and an inconsistent electrical contact between the particles and the GC supporting electrode (vide infra). We have highlighted the significant role of the area of electrical contact between inorganic particles and the support electrode in determining the electrochemical response in our recent experimental and modeling study^36^ of Li^+^ ion intercalation in LiMn_2_O_4_, and we would expect such effects to be important for the system herein, albeit for electrocatalysis. Under LD conditions, individual particles within the ensemble can significantly influence the overall OER activity, as implied by the results in Figure 3a.

Through a comprehensive investigation involving both particle ensembles (Figure 2) and individual particles (Figure 3), it was observed that there was increasing variation in the OER activity as the number of particles encapsulated by the meniscus decreased. Notably, when particles were probed individually, the variation in OER activity was at least an order of magnitude larger compared to measurements performed on the LD particle ensembles (i.e., 30-fold vs 3-fold, *vide supra*). Additionally, the value of θ had no impact on approaching consistent “bulk” OER activity when the particle number was low (Figure S6 in the Supporting Information). For instance, when the OER activity of multiple particles (three to four particles) was probed using an SECCM probe with a *d*_tip_ of 6 μm, and for θ values exceeding 0.5 (Figure S6c in the Supporting Information), the variation in OER activity between measurements remained significantly higher compared to those performed on larger ensembles with the probe with a *d*_tip_ of 55 μm (e.g., Figure 2). This indicates that at lower particle populations, the unique activities of the individual particles are revealed (e.g., Figure 3), leading to significant variation from measurement-to-measurement (i.e., from particle-to-particle).

On the other hand, in the case of HD particle ensembles with a comparably high particle population, the OER response approaches a state close to “bulk” activity, leading to high reproducibility in measurements across different particle ensembles. In other words, under HD conditions, the unique electrochemical activities of the individual particles become less important, with the ensemble instead producing a weighted average current density that is consistent from area-to-area. For β-Co(OH)_2_ particles, the transition from “individual particle” to “close-to-bulk” OER activity was observed for θ values above 0.27 (Figure S4 in the Supporting Information), corresponding to approximately 130 particles in an ensemble. It follows that SECCM measurements performed under these conditions are conceptually similar to a conventional “bulk” electrochemical measurement performed on a particle modified carbon electrode. These measurements thus reveal that there is a critical particle population required to represent a “bulk” material activity.

### Subparticle Imaging at the Nanoscale: Basal vs Edge Plane Activity

**Figure 4:**
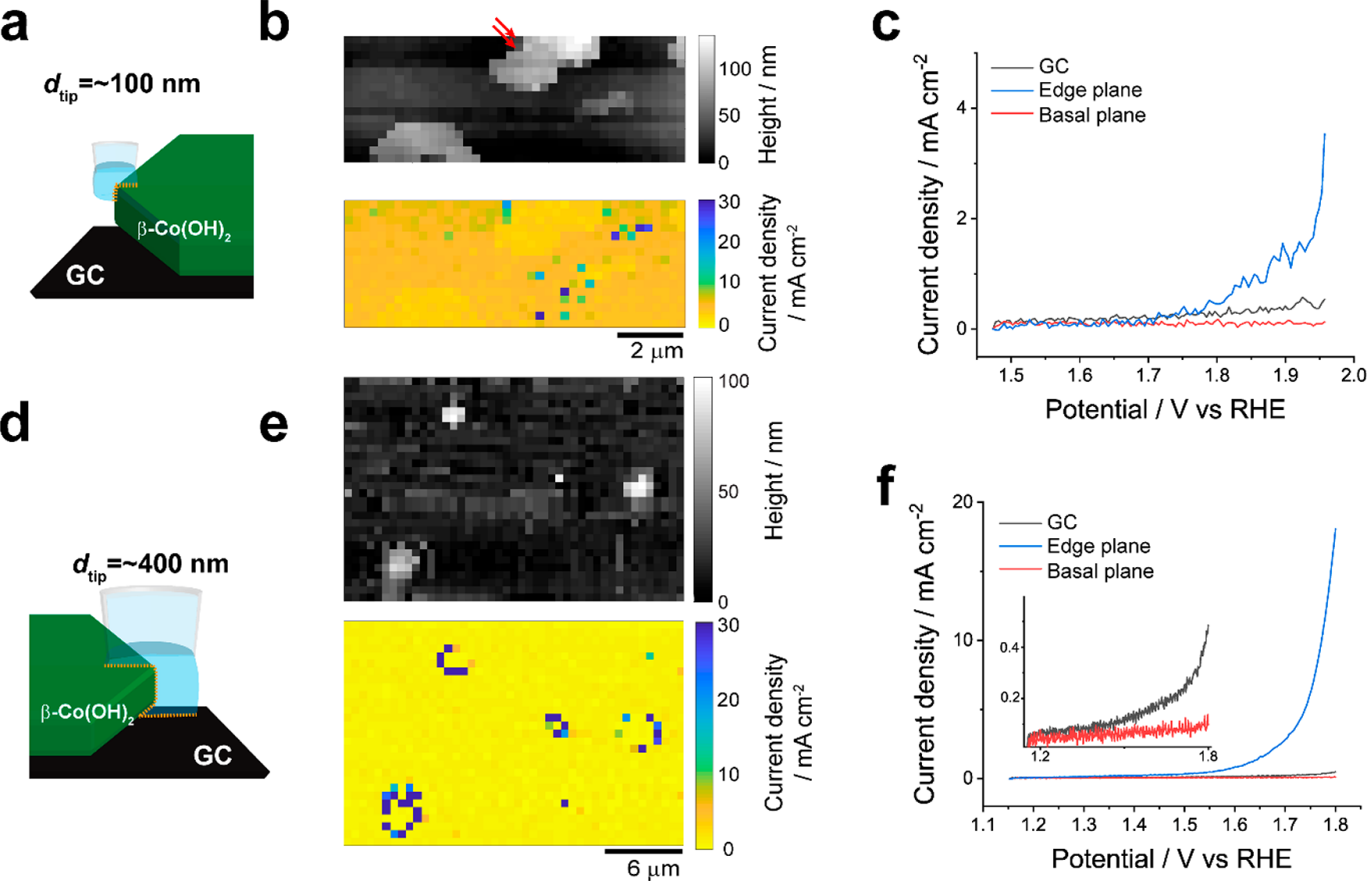
(a) Schematic of SECCM measurements carried out at the subparticle level (i.e., electrochemical mapping on a single particle) using nanopipet probes with *d*_tip_ values that are (a) comparable in size to (*d*_tip_ ≈ 100 nm) or (d) much larger than (*d*_tip_ ≈ 400 nm) the thickness (ca. 75 nm, assumed) of the β-Co(OH)_2_ particles. Note that the nanopipet probe depicted in (a) is not able to fully encapsulate the electrolyte|catalyst|support three-phase boundary. The electrolyte contact area at the edge of the particle is highlighted with a yellow dotted line. Topography and current density (1.8 V vs RHE) maps obtained with nanopipet probes of (b) *d*_tip_ = 120 nm and (e) *d*_tip_ = 440 nm. Representative LSVs (υ = 1 V s^–1^) from on GC (black) and β-Co(OH)_2_ particles on the basal (blue) and edge planes (red), obtained with nanopipet probes of (c) *d*_tip_ = 120 nm and (f) *d*_tip_ = 440 nm. The SECCM experiments were carried out in the voltammetric hopping mode by using dual-channel nanopipet probes filled with 0.1 M KOH.

Before delving into the *subparticle* variation in OER activity among individual particles, it was essential to assess whether SECCM is capable of distinguishing high activity on the edge plane through voltammetric electrochemical imaging with SECCM. As previously reported,^30,45^ voltammetric hopping mode SECCM enables the generation of synchronous topographical and electrochemical maps as a function of voltage, which can be transformed into an electrochemical movie, allowing *direct* and *unambiguous* structure–activity correlations. To directly correlate the OER activities with respect to the basal plane versus the edge plane, electrochemical maps of β-Co(OH)_2_ particles were acquired by using a probe significantly smaller than a single particle (*d*_tip_ = 120 nm; Figure 4a–c) employing a hopping distance of 200 nm. As noted above, dual-channel SECCM was deployed here for subparticle imaging due to the intrinsically low electrical conductivity of β-Co(OH)_2_ particles, prior to redox transformation (via oxidation).^50−52^ This configuration provided positional feedback (ionic current) independent of *i*_surf_, ensuring consistent meniscus–surface contact, regardless of the material conductivity (vide infra; Figure 1). Note that the current is normalized to current density using the probe orifice size, which was characterized through STEM or SEM images of the probe tip.

The SECCM scan (Movie S1 in the Supporting Information) performed with the 120 nm tip covered four individual particles as well as the remnants of a damaged particle, as depicted in the synchronously obtained topography map (Figure 4b). Overall, the SECCM electrochemical image (Figure 4b, obtained at 1.8 V vs RHE) did not exhibit a discernible difference in the OER activity between the basal plane and the edge plane. Only 2 out of 54 pixels that are measured around the periphery of the platelets (i.e., at the edge plane) showed slightly higher activity (Figure 4b; left side of the top middle particle) than the basal plane. Note that in both cases of elevated activity, the edge plane height (e.g., *z*-topography) is relatively lower than other areas of the platelet particle. The same is true for the 16 active pixels located near the remnants of the damaged particle, giving a total of 18 highly active active pixels out of 800 in Figure 4b (discussed in detail below).

To ensure simultaneous contact between the electrolyte, particle, and underlying GC support electrode, we increased the probe size to *d*_tip_ = 440 nm and performed voltammetric SECCM with a hopping distance of 600 nm. With the 440 nm probe, a distinct difference in OER activity between the basal plane and the edge plane on pristine particles (without obvious defects) was observed. The SECCM scan also covered all four individual particles (Figure 4e; Movie S2 in the Supporting Information), with the edge plane of each particle exhibiting significantly higher activity (Figure 4f). This is manifested in 35 of 46 pixels that were measured at the edge planes of these four individual particles (Figure 4e). Notably, the pristine basal plane displayed even lower electrochemical activity than the underlying GC support. This can be attributed to the intrinsically low electrical conductivity of β-Co(OH)_2_ and the limited ion transport directly through the CoO_2_ slabs on the {0001} oriented basal plane, which hinders the redox transformation of β-Co(OH)_2_ to CoO_*x*_H_*y*_, the OER-active form with higher electrical conductivity.^50−53^

Considering that the meniscus cell height is comparable to, or smaller than, the SECCM probe radius,^54,55^ and the average height of the edge plane is 75 nm, the 120 nm probe is only expected to capture the two-phase boundary at the edge plane, i.e., electrolyte|β-Co(OH)_2,edge_. In contrast, the 440 nm probe can capture the three-phase boundary, i.e., electrolyte|β-Co(OH)_2,edge_|GC (Figure 4a,d). This underscores the importance of the electrolyte|catalyst|support three-phase boundary for facilitating both ion and electron transport through the edge plane, thereby activating the OER. This is particularly true for materials of low intrinsic electrical conductivity (e.g., semiconductors) such as those based on transition-metal oxides^36^ or chalcogenides.^56^ To further support this idea, we refer back to the anomalous pixels in Figure 4b that exhibit a high OER activity when using the 120 nm probe. These pixels were exclusively observed at the edge plane of particles with a lower-than-average particle height (below 50 nm) and in the vicinity of the damaged particle, which also had a height below 50 nm. In such cases, the electrolyte meniscus from the 120 nm probe can encapsulate both β-Co(OH)_2,edge_ and GC or exposed defect sites in smaller β-Co(OH)_2_ debris (Figure S7 in the Supporting Information) and GC, leading to the observed high OER activity.

It is also pertinent to acknowledge the variation in the edge plane OER activity within individual platelet particles. Assessing 9 distinct particles, both herein (4 particles in Figure 4e) and in prior work (5 particles)^37^ reveals that between 30% and 95% of pixels situated on the edge plane (i.e., at the particle periphery) exhibit elevated OER activity compared to the basal plane. Also note that there is a significant variation in activity (current values) among edge plane pixels, for example, as clearly evident in Figure 4e (i.e., the activity “halos” around the individual particles are not of uniform color). This may be partially explained by the fact that a different amount of edge plane might be encapsulated by the meniscus from pixel to pixel. As explored below, also contributing to this is the fact that the platelet particle edges do not catalyze the OER to the same extent (e.g., edge-to-edge variability in activity). This result agrees with previous work that indicated significant heterogeneity in the average Co^*n*+^ oxidation state and volume expansion/contraction within a particle during the potential-dependent CoO_*x*_H_*y*_ phase transition.^37^

Note that while voltammetric hopping-mode SECCM^30^ offers a comprehensive current–voltage profile at each measurement point, its operation involves point-to-point measurements that are spatially independent (e.g., each pixel does not overlap with the area of the prior measurements). This inherently limits the lateral (*XY*) resolution to approximately the diameter of the employed probe. Thus, to obtain more information about the spatial distribution of activity over the electrolyte|catalyst|support three-phase boundary region, a constant distance scanning mode is employed below, which concurrently and continuously captures topography and activity for a fixed applied potential.^57^

### Subparticle Imaging at the Nanoscale: Particle-to-Particle Variations in Intraparticle OER Activity

The impact of physical contact between β-Co(OH)_2_ particles and the underlying GC support, as well as the influence of gross structural defects on the local OER activity of individual particles, were further investigated using a constant distance scanning mode with a dual-channel SECCM probe.^22,23^ The constant distance scanning mode involves the continuous movement of the meniscus across the surface while maintaining a fixed distance between the substrate surface and the glass pipet tip. This mode enables the acquisition of both topography and electrochemical activity with a higher lateral (*XY*) resolution compared to the voltammetric hopping mode above, despite utilizing a probe of the same dimensions.

To ensure comprehensive characterization, continuous line scans were performed across the individual particles. For this purpose, a probe with a *d*_tip_ of 440 nm was employed to ensure that the electrolyte meniscus simultaneously captured both the edge plane and the GC during the scan. The scanning protocol involved vertical oscillation of the probe in a sinusoidal wave pattern, generating an alternating current (*i*_AC_) to maintain a constant distance between the probe and the substrate surface (see Figure S8 in the Supporting Information). A fixed voltage of 1.87 V vs RHE, which exhibited the maximum contrast in electrocatalytic activity between the edge plane and basal plane of the particle, was applied to the substrate, while the SECCM probe was scanned over the particle. Instead of generating electrochemical maps or movies, two continuous line profiles were obtained: one representing the electrochemical properties (i.e., current density) and the other representing the topography (i.e., *Z*-position), both correlated to the lateral probe position (i.e., *X*-position). Note that the current is normalized to the current density based on the probe size.

**Figure 5:**
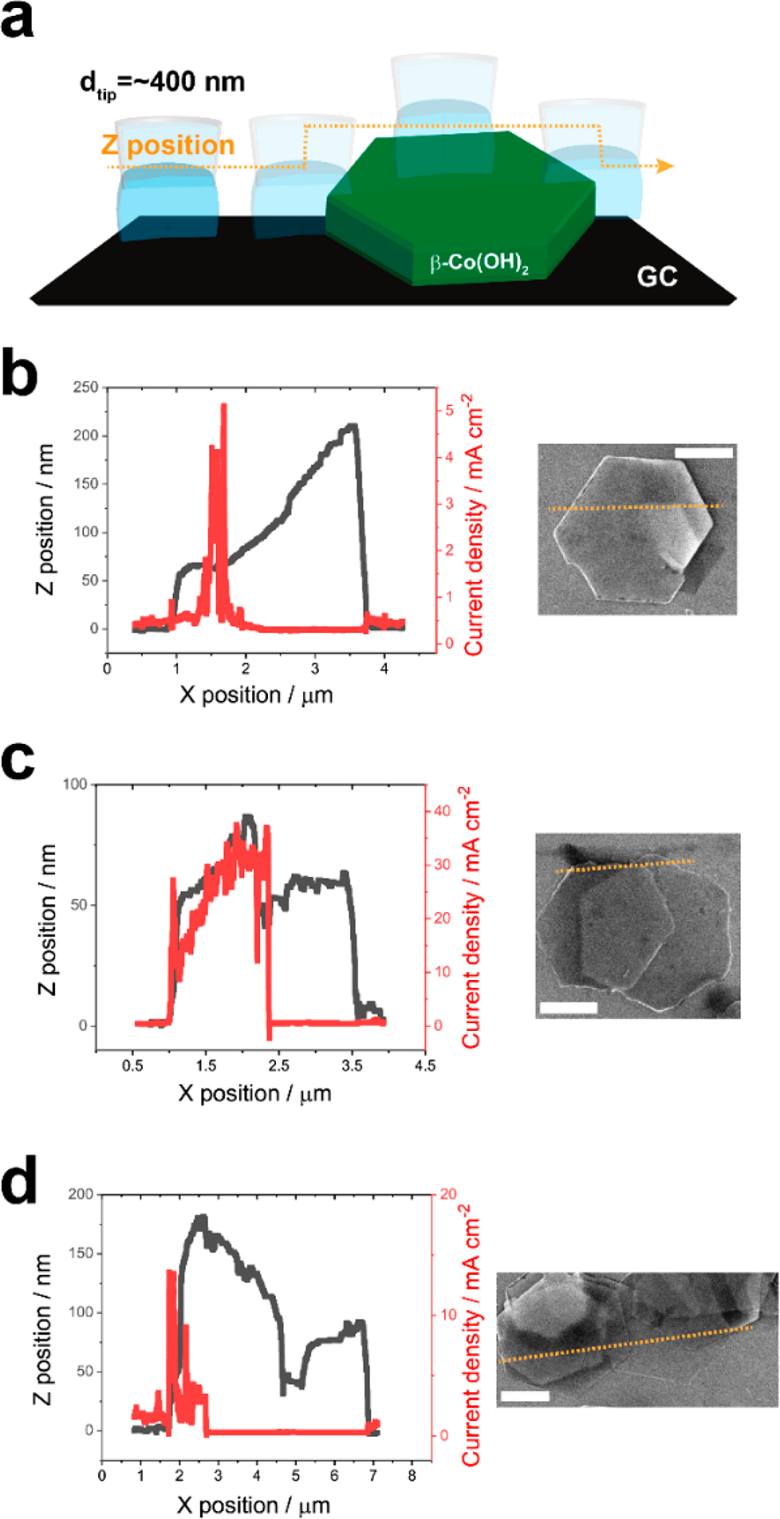
(a) Schematic of the constant distance scanning mode of SECCM, carried out with a dual-channel nanopipet probe with *d*_tip_ ≈ 400 nm. Line scan profiles of (b–d) current density (solid red trace; *V*_surf_ = 1.87 V vs RHE) and topography (solid black trace) as a function of *X*-position on (b) a single β-Co(OH)_2_ particle and (c and d) stacks of β-Co(OH)_2_ particles. Colocated SEM images are shown on the right of each plot. The SECCM experiments were carried out in the constant distance scanning mode (lateral translation speed 20 nm s^–1^) using a dual-channel nanopipet probe with *d*_tip_ = 440 nm, filled with 0.1 M KOH. Note that both *Z*-position and current are recorded synchronously during the measurements and the probe scanned the β-Co(OH)_2_ particles from left to right (orange dotted line in SEM images). All scale bars are 1 μm.

This approach allowed for a detailed analysis of the electrochemical and topographical characteristics along the scanned line across the particle (Figure 5). The high OER activity specifically at the edge plane of the β-Co(OH)_2_ particles was initially demonstrated through SECCM line profiles using a constant distance scanning protocol on two individual pristine particles (Figure S9a,b in the Supporting Information).^37^ The line scan profiles showed a clear contrast in the OER activity between the edge plane and basal plane, consistent with the electrochemical map presented in Figure 4e. Notably, as the probe crossed over an edge plane on a particle, a continuous region of elevated OER activity was observed, providing additional evidence of the selective electrocatalytic activity at this structural motif (i.e., {112̅0} and {101̅0} edge plane facets).

As proposed above, the establishment of a direct physical (electrical) contact between nanocatalyst and the supporting electrode is of paramount importance for achieving electrocatalytic turnover.^15,16,58,59^ The influence of insufficient physical (electrical) contact between the particles and the GC support on the OER activity is evident from the distinct current–topography behavior observed during line scanning, as shown in Figure 5. For example, Figure 5b illustrates the case where the OER activity is initially observed at the edge of the particle but diminishes as the meniscus cell passes over the region where the particle is lifted from the GC support. This observation highlights the crucial role of physical contact in sustaining the OER activity. Disruption of the three-phase boundary, involving interactions among the particle, the electrolyte, and the supporting electrode, due to incomplete contact leads to an apparent loss of OER activity. Note that although the “peak” in current density in Figure 5b is apparently offset from the location of the particle edge, it still takes place within one probe diameter (i.e., *within* 440 nm from the edge), meaning that the electrolyte|catalyst|support three-phase condition is still met.

The significance of the three-phase boundary is further supported by observations of particles stacked on top of each other, as illustrated in Figure 5c. When the SECCM meniscus cell crosses over the edge plane of the top-stacked particle (i.e., left particle in Figure 5c), the electrochemical line profile exhibits full OER activity, despite the slight lifting of the particle from the GC support caused by the bottom-stacked particle. (Note that in all cases, the line scans were performed from left to right). However, when the probe lands on the basal plane of the bottom-stacked particle (i.e., right particle in Figure 5c), the current density drops close to zero. This observation highlights that the electrochemically active part of one particle (i.e., top-stacked particle) does not influence adjacent or connected particles (i.e., bottom-stacked particle). Again, in line with the discussions above, the CoO_2_ slabs of the intact basal plane are not conducive to either electron or ion transport, hindering the OER activity on the meniscus contacted area of the bottom-stacked particle. Similar observations are made with multistacked particle ensembles (Figure 5d), where the OER activity is only observed on the topmost stacked particle where the electrolyte|catalyst|support three phase boundary requirement is met. In other words, the particles underneath the top-stacked particle do not show OER activity, even at their exposed edge planes, due to lack of synchronous contact with electrolyte and the underlying GC support electrode (required to establish the aforementioned three-phase interface).

These findings underpin the critical role of establishing a direct physical (electrical) contact between individual particles and the supporting electrode for achieving the OER activity in β-Co(OH)_2_ particles. Ensuring an intimate electrolye|catalyst|support three-phase boundary is essential for facilitating electrochemical reactions and maximizing the electrocatalyst utilization. The presence of any electrical resistance or disruption in the physical contact can significantly impede charge transport and compromise the observable OER activity.

**Figure 6:**
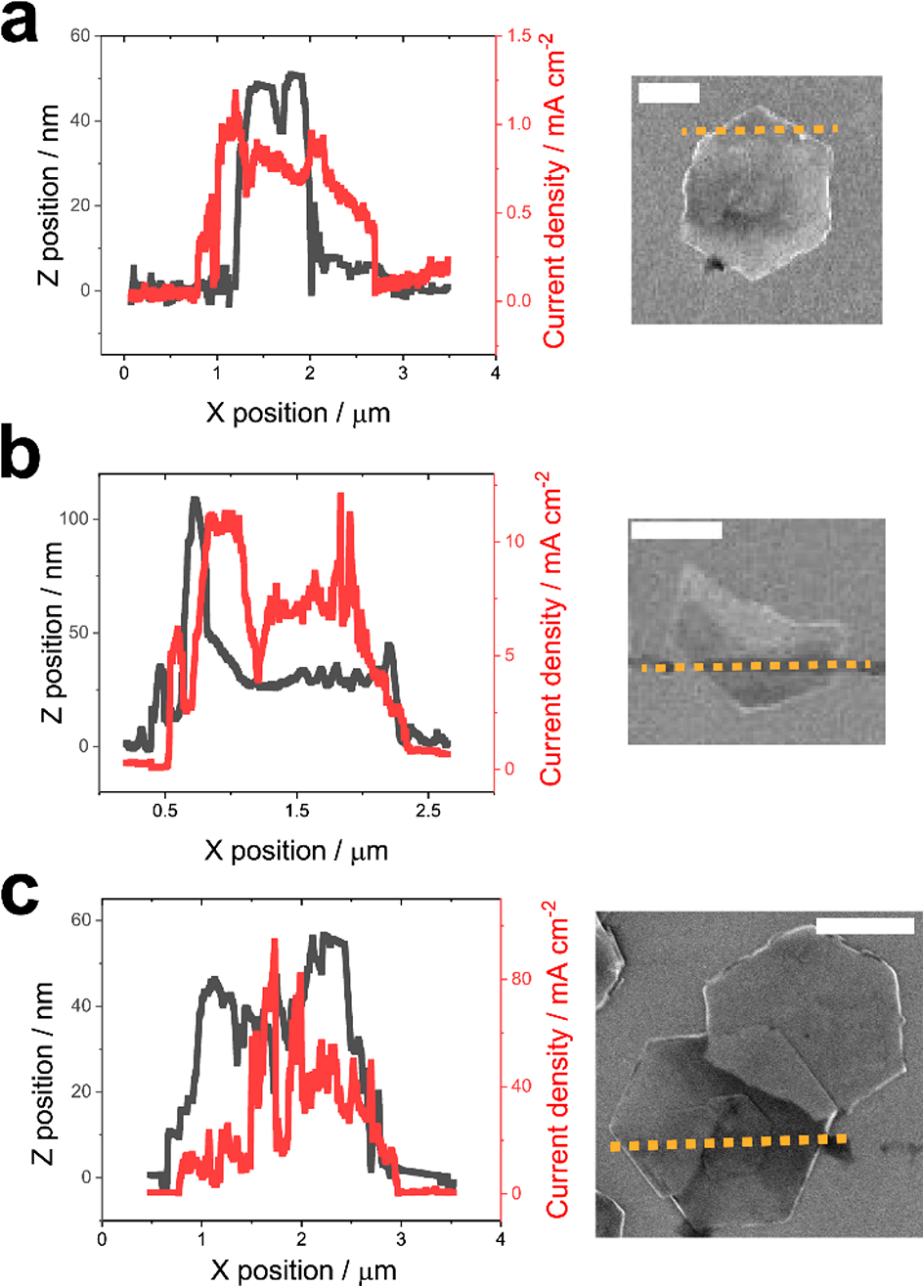
Line scan profiles of current density (solid red trace) and topography (solid black trace) as a function of *X*-position, obtained across three representative “defective” β-Co(OH)_2_ particles. The respective particles are (a) misshapen (*V*_surf_ = 1.64 V vs RHE), (b) fragmented (*V*_surf_ = 1.74 V vs RHE) and (c) contain a screw dislocation (*V*_surf_ = 1.87 V vs RHE). The SECCM experiments were carried out in constant distance scanning mode (lateral translation speed 20 nm s^–1^) using a dual-channel nanopipet probe with *d*_tip_ = 440 nm, filled with 0.1 M KOH. All scale bars are 1 μm.

Interestingly, when the β-Co(OH)_2_ particles exhibit gross structural defects (e.g., cracks and splits), the basal plane can exhibit high OER activity, which can be observed using SECCM line scans in the constant distance mode. In Figure S7b in the Supporting Information and Figure 6, TEM and SEM images reveal the presence of damaged particles among the population of particles on the GC substrate. The cracks and splits in these particles may have occurred during the electrode preparation process when suspending (e.g., during ultrasonication), casting, and immobilizing the particles on the GC substrate (refer to Methods).^60,61^ These gross structural defects within the particles appear to facilitate charge (i.e., ion + electron) transport within the entire particle, leading to non-edge-selective OER activity.

In Figure 6a, the particle exhibits a clear shape deformation, deviating from the typical hexagonal particle shape. Additionally, the basal plane of the particle exhibits high roughness. Although not apparent in the SEM image, the *Z*-position profile also reveals the presence of a crevice (of ca. 10 nm, located at an *X*-position of ca. 2 μm) within the basal plane. Across the full line scan, the particle exhibits an OER activity, including at both the edge and basal planes. Gross structural defects, emergent on the basal plane, induced by particle fragmentation also frequently support OER activity (Figure 6b). In addition, even relatively subtle surface defects, such as screw dislocations, can impact the OER activity in the basal plane (Figure 6c). Notably, distinct from particles with gross defects, which result in a relatively uniform OER activity across the entire particle surface on the scale of the measurement (Figure 6a,b), local defects such as screw dislocations lead to a “spike” in current density localized around the defect site (Figure 6c). These screw dislocations, observed occasionally in β-Co(OH)_2_ particles, expose basal steps with higher surface energy than the basal terrace, potentially resulting in higher electrocatalytic activity.^62,63^ Clearly, the presence of structural defects on the particle is crucial in “activating” the OER on the basal plane and influencing the overall OER activity of the particle.

Evidently, in accord with the *single-particle* measurements above, *subparticle* measurements, in the form of activity line profiles, reveal significant particle-to-particle variations in OER activity. Although line profiles do not probe an entire particle, alongside SEM analysis, they have revealed the importance of electrical/physical contact between the material and the supporting electrode as well as the presence of defects on the basal plane in the overall particle activity. These observations underscore the significance of *multiscale* SECCM, as it offers highly versatile and truly localized electrochemical analysis from the *subparticle* to *single-particle* to small particle ensemble levels, allowing for a comprehensive understanding of the factors influencing OER activity across length and time scales.

## Conclusions

In conclusion, this study demonstrated the feasibility of multiscale SECCM by adjusting the tip orifice size and scanning protocols. The dimensions of the electrochemical cell were determined by the size of the electrolyte meniscus at the probe tip and by employing probes of various sizes, ranging from 55 μm to 120 nm in diameter; the analysis of β-Co(OH)_2_ platelet particles was carried out at different length scales.

While ensemble particle analysis (ca. 40–250 particles) revealed OER activities that are broadly consistent with conventional “bulk” macroscopic measurements, unique behaviors were observed which depended on the number of particles accessed. A high particle population, greater than ca. 130 particles, exemplified by HD particle ensembles, closely approached bulk activity in the OER response with low ensemble-to-ensemble variability. Conversely, particle ensembles with smaller particle populations (i.e., LD conditions) exhibited wider deviations from bulk behavior with decreasing population size, which manifested in more ensemble-to-ensemble variability (i.e., ca. 3-fold difference in catalytic current density from lowest to highest activity, *N* = 12). At the *single-particle* level, even more significant variations in the OER activity were observed, e.g., ca. 30-fold difference in current density from the least to most active particles (*N* = 22). While we attempted to correlate variations in activity with the size and structure of β-Co(OH)_2_ particles via identical-location multimicroscopy analysis with AFM and SEM, no direct correlation was found between the OER activity and the particle size, basal plane roughness, or exposed edge plane area. Factors other than size and structure, such as the low intrinsic electrical conductivity of the β-Co(OH)_2_ material and inconsistent physical (electrical) contact between particles and the GC support, were identified as likely contributing factors for the observed variations.

Electrochemical mapping at the subparticle level directly confirmed that the contact among particles, the electrolyte, and the GC support (i.e., electrolye|catalyst|support three-phase boundary) is crucial for facilitating ion and electron transport, thereby selectively activating the OER at the edge plane of pristine β-Co(OH)_2_ particles. The need for direct physical (electrical) contact between the particle and the supporting electrode was further supported by line scan profiles obtained in the constant distance scanning mode, which also demonstrated that the presence of gross structural defects on the basal plane influences local catalytic activity within the particle, showing that the basal plane can be OER active.

Overall, this study highlights the importance of considering multiple factors beyond particle size and morphology when investigating the electrochemical activity of materials with low intrinsic electrical conductivity (e.g., electrocatalysts, battery materials, etc.). These results have dramatic implications for the utilization of catalyst layers in the membrane electrode assemblies of electrolyzers, where deviations in coverage and/or pore structure between the catalyst layer and the porous transport layer/gas diffusion layer means there will unavoidably be a high degree of inactive and underutilized catalyst mass for low-conductivity materials. Overcoming this limitation necessitates the development of intrinsically conductive electrocatalysts or corrosion resistant conductive additives that can operate under the harsh conditions encountered during the OER.

## Methods

### Chemical Reagents and Electrode Materials

Potassium hydroxide (KOH, semiconductor grade, Sigma-Aldrich), potassium chloride (KCl, 99.5%, Honeywell, Germany), dodecane (>99%, Merck), tetrahydrofuran (THF, ≥99.9%, Sigma-Aldrich), hexamethylenetetramine (HMT, ≥99.0%, Sigma-Aldrich) and cobalt(II) chloride hexahydrate (CoCl_2_·6H_2_O, 98%, SigmaAldrich) were used as supplied by the manufacturer. All aqueous solutions were prepared with ultrapure deionized water (DI water, resistivity 18.2 MΩ cm at 25 °C).

The β-Co(OH)_2_ particles were synthesized as previously described.^38^ Briefly, a 400 mL volume of aqueous 45 mM HMT solution was heated to 85 °C (with magnetic stirring) under an atmosphere of high-purity nitrogen (N_2_). A 3.5 mmol portion of CoCl_2_·6H_2_O was dissolved in 20 mL of DI water, added dropwise to the HMT solution, and then allowed to reflux for 5 h. The stirring was then ceased before allowing the solution to cool to room temperature under the N_2_ atmosphere. The precipitate was collected through centrifugation and washed subsequently with DI water and anhydrous ethanol before finally being dried overnight at 80 °C. An “ink” of β-Co(OH)_2_ was prepared by suspending 2 mg of the dry precipitate in 3 mL of THF by gentle sonication for no more than 1 min. GC-supported β-Co(OH)_2_ electrodes were prepared by drop-casting 3 μL of the ink onto a freshly cleaned GC plate (HTW-Germany). After the ink was allowed to dry on the GC surface, the drop-cast area was stamped gently with a clean polydimethylsiloxane (PDMS) block to remove loosely bound, agglomerated particles. The surface of the prepared substrate was then covered with a thin layer of dodecane, a nonpolar and very low dielectric medium, which prevented leakage of the SECCM meniscus cell during scanning experiments (vide infra).^28,41,42^

Ag/AgCl QRCEs were prepared by anodizing Ag wire (125 μm diameter, 99.99%, Goodfellow, U.K.) in an aqueous saturated KCl solution. The Ag/AgCl QRCEs possessed a reference potential (calibrated before and after each SECCM experiment) of 0.238 ± 0.004 V vs Ag/AgCl (3.4 M KCl) in 0.1 M KOH, which is stable on a time scale of several hours.^64^ Note that in all SECCM experiments, the reference potential was converted to the reversible hydrogen electrode (RHE) scale, as previously reported.^45^

### Probe Fabrication

Four different types of pipet probes were used for SECCM. Single-channel pipet probes, with diameters of ca. 55 and 6 μm, were fabricated from filamented borosilicate glass capillaries (OD 1.0 mm, ID 0.5 mm, World Precision Instruments Inc., USA), using a PC-10 Dual-Stage Glass Micropipette Puller (Narishige, Japan). The dual-channel pipet probes, with diameters of either ca. 440 or 120 nm, were fabricated from filamented quartz glass theta capillaries (OD 1.2 mm; ID 0.9 mm, Friedrich & Dimmock Inc., USA), using a P-2000 CO_2_-laser puller (Sutter Instruments, USA). After pulling, the probes were backfilled with 0.1 M KOH solution using a MicroFil Syringe (World Precision Instruments Inc., USA), before adding a thin layer of silicone oil (DC 200, Sigma-Aldrich) on top to minimize evaporation from the back of the pipet during prolonged scanning, as previously reported.^45^ Ag/AgCl QRCEs were then inserted into each barrel, through the layer of silicone oil, into the 0.1 M KOH solution to finalize the SECCM probe, rendering it ready for use. After scanning, the probes were carefully emptied and rinsed with DI water (using a clean MicroFil syringe) before imaging the tip with SEM.

### Scanning Electrochemical Cell Microscopy

Local electrochemistry was carried out in the SECCM format on a home-built scanning electrochemical probe microscopy (SEPM) workstation at the University of Warwick, UK, as previously reported.^21−23,32,54^ In this configuration, the constructed SECCM probe (i.e., filled pipet equipped with QRCE(s), vide supra) was mounted on a *z*-piezoelectric positioner and the GC-supported β-Co(OH)_2_ working electrode (WE) was placed on an *xy*-piezoelectric positioner. All piezoelectric positioners were purchased from Physik Instrumente, Germany. When using a double-barrel probe (vide supra), a bias potential (*V*_2_) of 0.05 V was applied between the QRCEs to induce a DC ion current (*i*_DC_) between the barrels, used for probe positioning, independent of the local activity and/or electrical conductivity, as previously reported.^4^ The SECCM probe was positioned initially above the WE surface using coarse *xy*-micropositioners (M-461-XYZ-M, Newport, USA) and subsequently lowered to a near-surface position using a stepper motor (Picomotor, Newport, USA), with the aid of an optical camera (PL-B776U, PixeLINK, Canada). SECCM was operated in two modes: *voltammetric hopping mode* and *constant-distance mode*.

In the *hopping mode* (i.e., a series of fixed tip positions in contact with the surface, scanning voltage), the pipet probe approached the WE surface in a predefined grid pattern of locations and, upon each landing, an electrochemical measurement was made, so as to obtain a data set that could be used to create spatially resolved voltammetric (*i*–*E*) “images” of the substrate surface. Surface current (*i*_surf_) and *i*_DC_ feedback were employed with single-barrel and double-barrel probes, respectively. A single “hop” of a scanning experiment involved (i) approaching the pipet probe to the WE surface until meniscus contact was made, detected when either the *i*_surf_ or *i*_DC_ feedback threshold was reached, stopping further *z*-translation, (ii) recording a linear-sweep voltammogram (LSV), localized to the confined area defined by the meniscus–WE contact, (iii) retracting the probe from the WE surface, and (iv) translating the probe in *xy*-space, ready for the next “hop”. Note that the pipet itself did not physically contact the substrate at any point during scanning. In addition, the final position of the *z*-piezoelectric positioner at approach was used to construct a topographical map of the GC-supported β-Co(OH)_2_ WE surface. Considering the tip resistance of the probe with *d*_tip_ = 55 μm and the maximum current measured, which remains below 100 nA, the *iR* drop is calculated to be at most ∼18 mV. For probe sizes below 55 μm, such as the probes with *d*_tip_ = 6 μm, 440 nm, and 120 nm, the calculated *iR* drop is <10 mV. *iR* drop on this scale does not significantly affect the shape of the measured *i–V* curves or the conclusions drawn from them. It is also important to note that the voltammograms are compared within a specific probe size. Thus, the *iR* drop has not been corrected for, herein.

In the *constant-distance mode* (i.e., scanning tip), after initial meniscus landing on the GC-supported β-Co(OH)_2_ WE surface (as described above), the pipet probe was rastered in *xy*-space at a fixed potential while maintaining a constant tip–substrate separation. This was achieved by modulating the *z*-position of the double-barrel nanopipet probe (amplitude 40 nm, frequency 327 Hz) by implementing an AC signal generated by a lock-in amplifier (SR830, Stanford Research Systems, USA), and the resulting AC ion current (*i*_AC_) was picked out for precise probe positioning (set point), as previously reported.^21−23,32,54^ In essence, by maintaining *i*_AC_ at a constant value during lateral scanning, the tip–substrate distance was fixed, allowing line scans of electrochemical activity (*i*_surf_, measured at a static potential) and topography (*z*-height) to be recorded synchronously.

The SEPM setup was situated on a vibration isolation platform (25BM-10, Minus K, USA) and placed within an aluminum Faraday cage equipped with heat sinks and acoustic foam. This configuration minimizes mechanical vibration, electrical noise, and thermal drift during prolonged scanning.^65^ The QRCE potentials were controlled, with respect to ground, with a home-built bipotentiostat, and the current flowing at the GC-supported β-Co(OH)_2_ WE (i.e., *i*_surf_), held at a common ground, was measured with a home-built electrometer. When using a single-barrel pipet probe, the potential of the working electrode surface was *V*_surf_ = −*V*_1_. When using a double-barrel pipet probe, *V*_surf_ = −(*V*_1_ + *V*_2_/2). All signals were outputted (i.e., *V*_1_, *V*_2_, etc.) or measured (i.e., *i*_surf_, *i*_DC_, *i*_AC_, etc.) synchronously every 4 μs and averaged in 512 blocks to give an effective data acquisition rate of 4 × (512 + 1) = 2052 μs, where one extra iteration was used to transfer the data to the host computer. Instrumental control and data acquisition were carried out using an FPGA card (PCIe-7852R) controlled by a LabVIEW 2016 (National Instruments, USA) interface running the Warwick Electrochemical Scanning Probe Microscopy (WEC-SPM, www.warwick.ac.uk/electrochemistry) software.

### Surface Characterization

Optical microscopy (OM) was performed on a BH2-UMA light microscope (Olympus, Japan). SEM imaging was carried out on a GeminiSEM 500 system (Zeiss, Germany). TEM imaging was carried out in bright-field mode using an aberration-corrected Titan ETEM 80–300 (FEI Company, USA) operated at 300 kV under an ultrahigh vacuum. AFM was carried out in tapping mode using silicon probes with a spring constant of 3 N m^–1^, as per the manufacturer’s specifications (RFESP, Bruker, Germany).

### Data Analysis and Processing

After acquisition, the raw SECCM data were processed using the Matlab R2020a (Mathworks, USA) software package. Data plotting was carried out by using the Matlab R2020 and OriginPro 2019b (OriginLab, Northampton, MA) software packages. AFM image processing was carried out with the scanning probe image processing software package (SPIP version 6.0.14, Image Metrology, Denmark). OM and SEM images of the GC-supported β-Co(OH)_2_ electrodes were analyzed using the ImageJ (NIH, USA) software package. The obtained SEM images were further processed by generating binary images and utilizing the automated particle counting function, readily available in the open-source ImageJ software, to estimate the coverage of β-Co(OH)_2_ nanoplates on the GC surface as well as their projected area. Note that all electrochemical maps and movies are presented without any data interpolation.

## Supporting Information Available

Physical and electrochemical stability of β-Co(OH)_2_ particles in oil, additional analysis of particle ensembles and individual particles, movie captions, and SECCM line scan protocols and additional line scan profiles of particles (PDF)
Spatially resolved electrochemical (current–voltage) movie (Movie S1; 40 × 15 pixels over a 10 × 2.75 μm^2^ area) obtained with the voltammetric (υ = 1 V s^–1^) SECCM configuration, visualizing OER activity of β-Co(OH)_2_ particles on the GC supporting electrode, corresponding to Figure 4b in the main text (AVI)
Spatially resolved electrochemical (current–voltage) movie (Movie S2; 44 × 29 pixels over a 26.4 × 17.4 μm^2^ area) obtained with the voltammetric (υ = 1 V s^–1^) SECCM configuration, visualizing OER activity of β-Co(OH)_2_ particles on the GC supporting electrode, corresponding to Figure 4e in the main text (AVI)

## Supplementary Material

nn3c06335_si_005.pdf

nn3c06335_si_002.avi

nn3c06335_si_003.avi
