# Supplementary material for: Multiscale Analysis of Electrocatalytic Particle Activities: Linking Nanoscale Measurements and Ensemble Behavior
Source: ACS Nano. 2023 Oct 26;17(21):21493–505. doi: 10.1021/acsnano.3c06335 (PMC10655184; doi:10.1021/acsnano.3c06335)
Supplement: Supplementary file 1 — nn3c06335_si_005.pdf [file nn3c06335_si_005.pdf]

Multiscale Analysis of Electrocatalytic Particle Activities: Linking Nanoscale Measurements and Ensemble Behavior

Minkyung Kang,[1,2,†*] *Cameron L. Bentley,*[3,†] *J. Tyler Mefford,*[4] *William C. Chueh*[4] and *Patrick R. Unwin*[2*]

[†] Equally contributed

[1]School of Chemistry, The University of Sydney, Camperdown, 2006, NSW, Australia

[2]Department of Chemistry, The University of Warwick, Coventry CV4 7AL, UK

[3]School of Chemistry, Monash University, Clayton 3800 VIC, Australia

[4]Department of Materials Science and Engineering, Stanford University, Stanford, California 94305, USA

E-mail: minkyung.kang@sydney.edu.au (M.K.); p.r.unwin@warwick.ac.uk (P.R.U.)

Contents

Section S1. Physical and electrochemical stability of β-$Co(OH)_2$ particles in oil S2

Section S2. Additional analysis of particle ensembles S4

Section S3. Additional analysis of individual particles S7

Section S4. Movie captions...S10

Section S5. SECCM line scan protocols and additional line scan profiles of particles S11

References S13

Section S1. Physical and electrochemical stability of β-$Co(OH)_2$ particles in oil

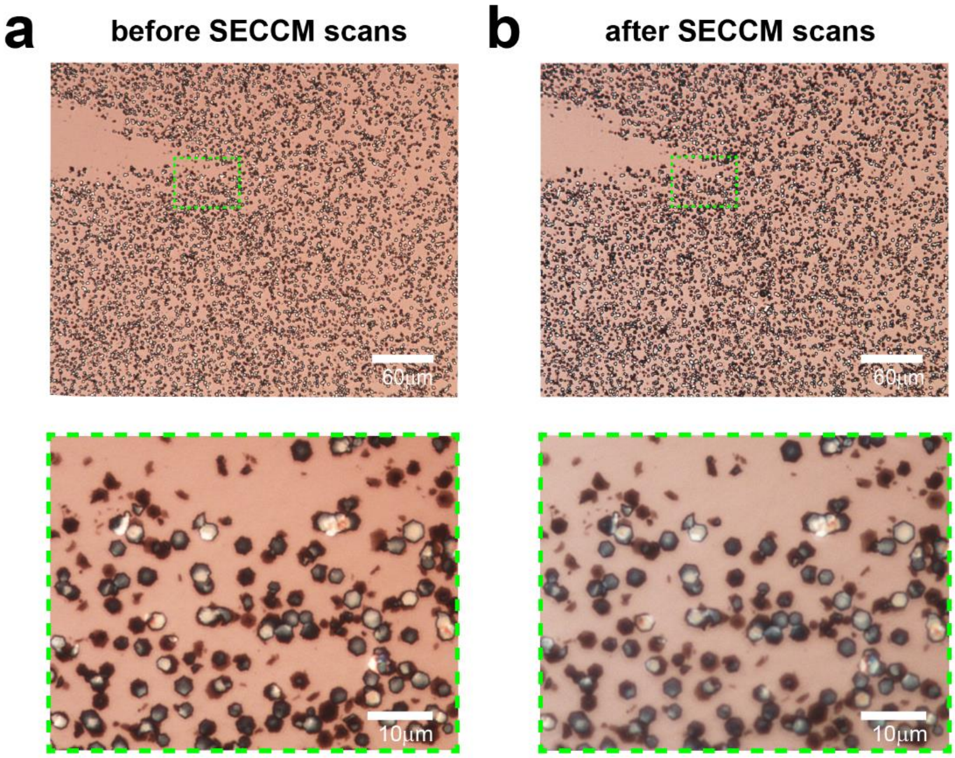

Figure S1. Optical microscopy images of β-$Co(OH)_2$ platelets particles on glassy carbon (GC) substrate, taken (a) before and (b) after SECCM experiments under an inert layer of dodecane. The area from which the higher magnification images (shown below) were obtained is highlighted with a dotted green box.

The optical microscopy images are visually identical, pre- and post- immersion under dodecane, confirming that the presence of the inert oil layer and/or the probe translocation during SECCM scans does not significantly affect the physical contact of β-$Co(OH)_2$ particles with the underlying GC support surface.

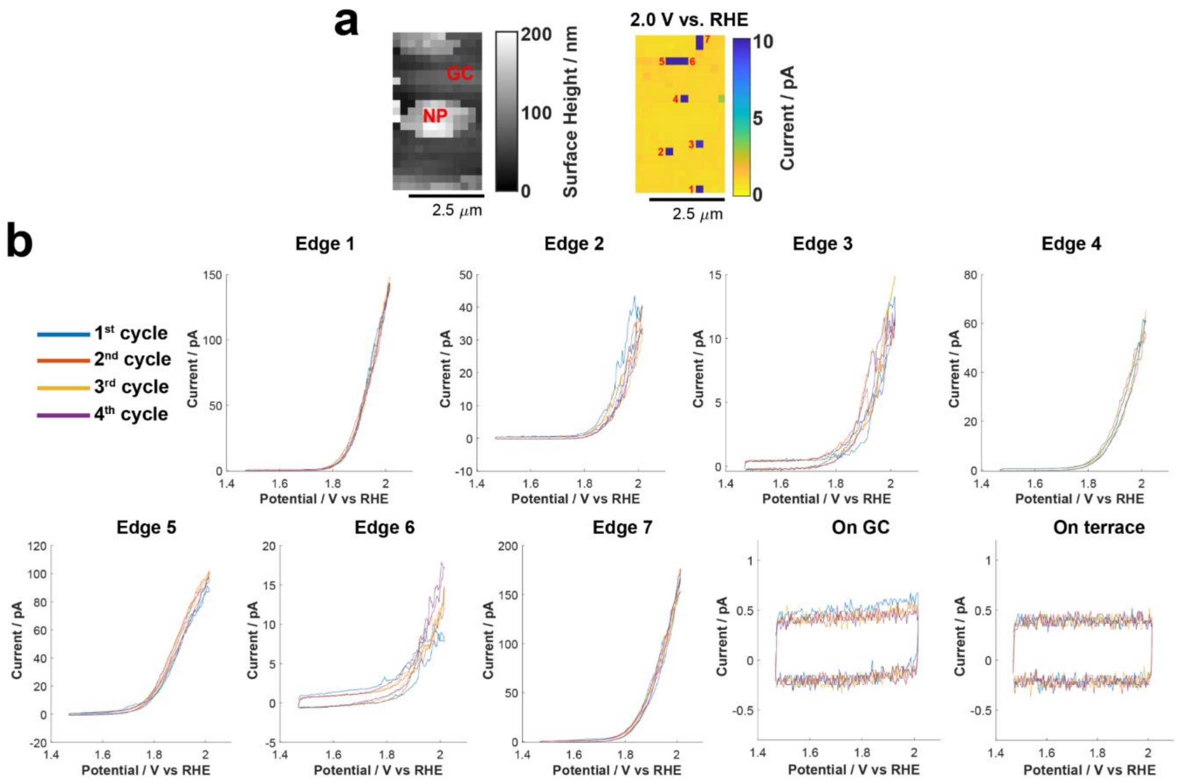

Figure S2. (a) Topography (left) and OER activity (right) maps of β-$Co(OH)_2$ particles on GC obtained from SECCM in the voltammetric hopping mode. A dual barrel SECCM probe (d_{tip} = 120 nm; hopping distance = 250 nm) filled with 0.1 M KOH was used, performing 4 cycles of cyclic voltammetry (CV; υ = 1 V s^{-1}) at each pixel. The OER activity map is a snapshot from the first CV cycle, taken at 2.0 V vs RHE, and the active pixels taken from the edge of the particles are annotated from 1 to 7 (blue; v*ide infra*). Note that not all of the edges appear to be OER active due to the inability to form a electrolyte|catalyst|support three phase contact (explored in the main text, Figure 5a). (b) CVs extracted from the annotated edges in (a), as well as from the terrace of the particles and GC.

As discussed in the main text, to confine the SECCM nanodroplet during the scanning in 0.1 M KOH (pH 13), a chemically inert layer of high-purity dodecane (>99.8%) was used. To ascertain whether the oil layer progressively "contaminates" the oxygen evolution reaction (OER) active site, multiple cyclic voltammograms (CVs) were recorded at each pixel. Evidently, the CVs extracted from active pixels exhibit over cycle-independent OER activities, suggesting that surface contamination by the adsorption of adventitious organic compounds is not an issue in the presence of the inert oil layer, in line with previous reports.[1-4]

Section S2. Additional analysis of particle ensembles

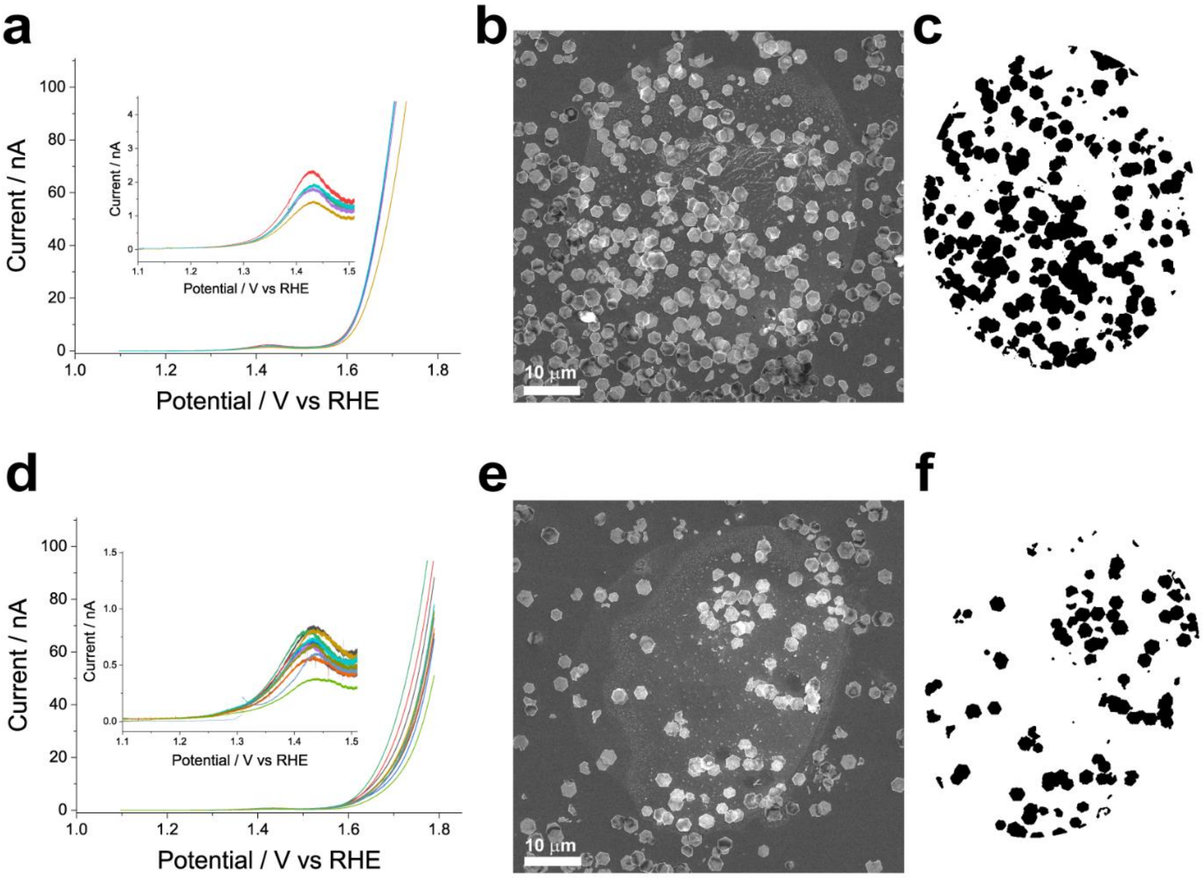

Figure S3. Comparison of the OER activity between high density (HD) and low density (LD) particle ensembles, recorded using a single channel SECCM probe (d_{tip} = 55 μm) containing 0.1 M KOH. (a) Linear sweep voltammograms (LSVs) of HD (N=6) and (d) LD (N = 12) particle ensembles obtained with a voltammetric scan rate (υ) of 10 mV s^{-1}. Note that the y-axis represents raw current data (i.e., not normalized to current density, as in main text, Figure 2). Representative scanning electron microscopy (SEM) images of micro-droplet footprints on (b) HD particle ensembles and (e) LD particle ensembles, acquired after SECCM measurements. Images of the projected area of (c) HD and (f) LD particle ensembles (black shapes) on a GC substrate (white background) within the droplet, derived from the acquired SEM images (b and e, respectively). (c) and (f) correspond to particle coverages (θ) of ca. 0.4 and 0.17, respectively.

Note that in this study, the terms 'HD' and 'LD' refer to high density and low density particle ensembles, respectively. As discussed in the main text, HD corresponds to a particle coverage (θ) within the microdroplet footprint of >0.3, whereas LD corresponds <0.22. These values are defined relative to the mean θ value of 0.26, taken from 28 measurements.

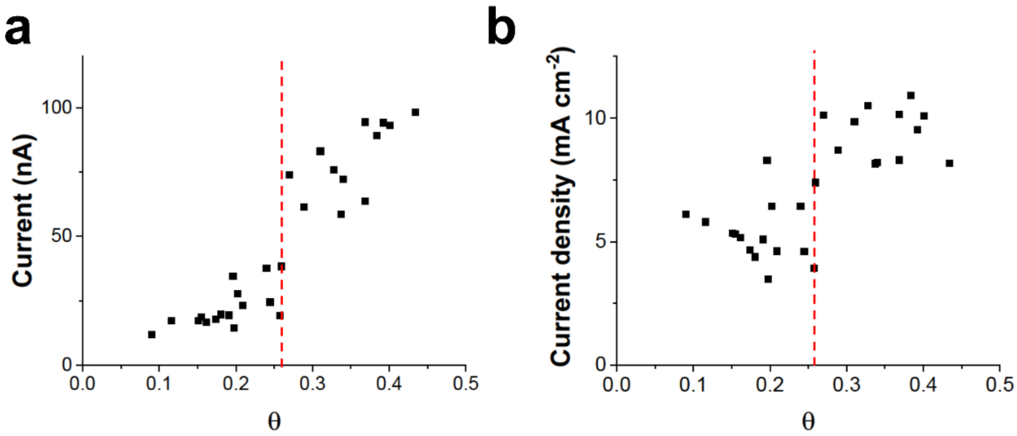

Figure S4. (a) Current and (b) current density at 1.71 V vs RHE as a function of particle coverage (θ) on the GC electrode (*N* = 28). The average particle coverage of θ = 0.26 is represented by a red-dotted line.

Table S1. Mean values and standard deviation (S.D.) of slope 1 and slope 2 (Figure 2 in the main manuscript), evaluated from Tafel analysis of the HD and LD β-Co(OH)$_2$ particle ensembles.

	HD particle ensembles		LD particle ensembles	
	slope 1 (mV dec^{-1})	slope 2 (mV dec^{-1})	slope 1 (mV dec^{-1})	slope 2 (mV dec^{-1})
Mean	67	135	92	131
S.D.	3	3	10	6

Section S3. Additional analysis of individual particles

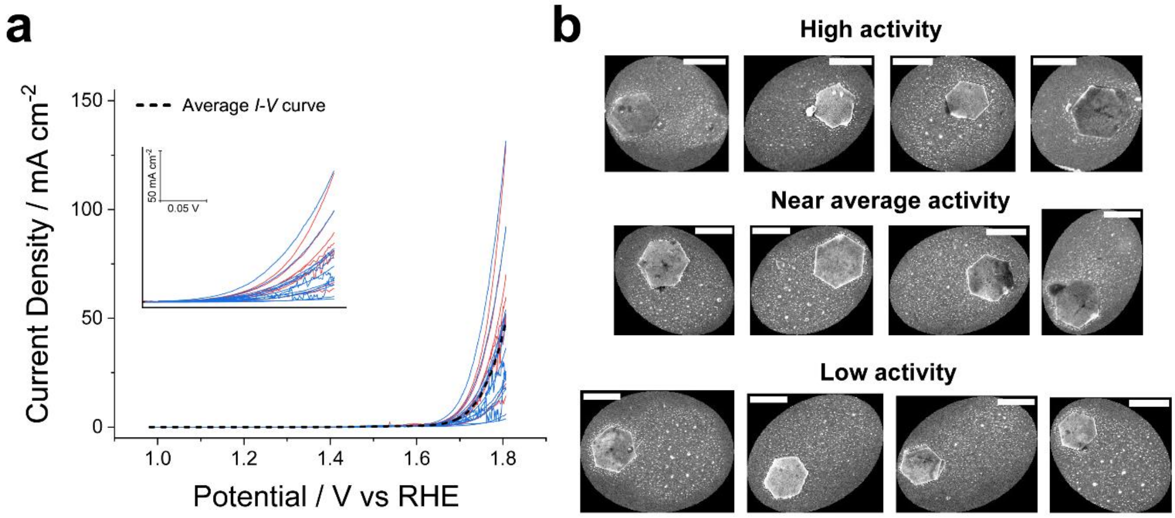

Figure S5. (a) LSVs of 22 individual β-$Co(OH)_2$ particles and their average I-V curve (dotted line), obtained using a single channel SECCM probe with d_{tip} = 6 μm, filled with 0.1 M KOH, at scan rates (υ) of 50 (Blue) or 100 (Red) mV s^{-1}. The inset was recreated using the original data from Figure S5a, and the potential range was adjusted to span from 1.6 V to 1.81 V. (b) SEM images correspond to the degree of OER activity. Scale bars: 2 μm.

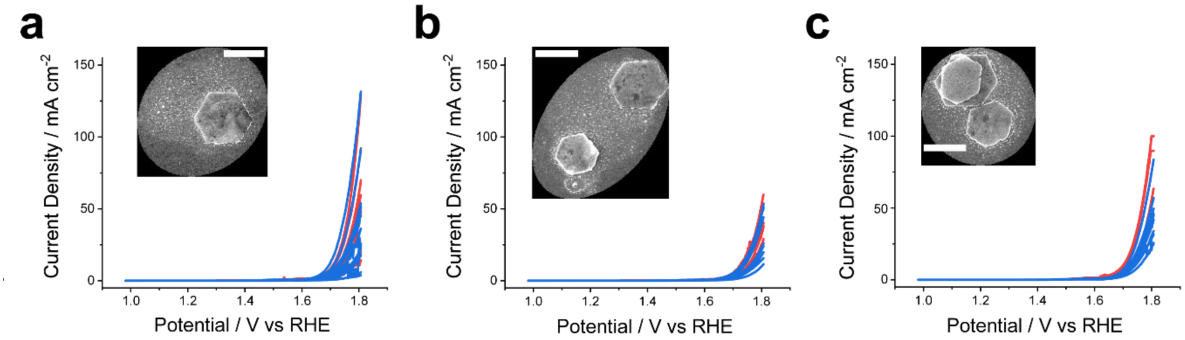

Figure S6. Individual LSVs of (a) single particles, (b) double particles, and (c) multiple (e.g., three to four) particles, along with representative SEM images for each case. LSVs were recorded at scan rates of 50 (blue) or 100 (red) mV s^{-1}.

The OER activity, as determined by comparing the current density at 1.78 V vs. RHE, does not scale linearly with the absolute particle coverage, the number of particles within the confined area, or the duration of measurements (i.e., voltammetric scan rate) in the case of single or small ensemble (i.e., <5) measurements. Additionally, the variation in OER activity between measurements is considerably greater compared to particle ensembles (e.g., main text, Figure 2).

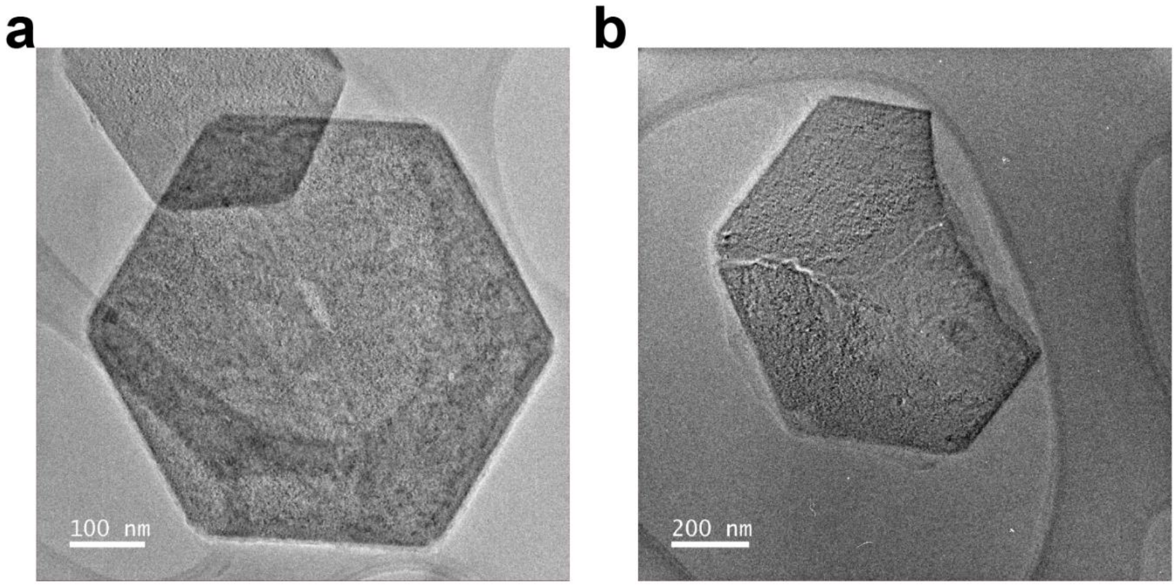

Figure S7. Representative transmission electron microscopy (TEM) images showing (a) relatively pristine and (b) fragmented β-$Co(OH)_2$ particles.

Section S4. Movie captions

Movie S1. Spatially-resolved electrochemical (current−voltage) movie (40 × 15 pixels over a 10 × 2.75 μm^2 area, hopping distance 250 nm) obtained with the voltammetric (υ = 1 V s^{-1}) hopping mode SECCM configuration (shown in the main text, Figure 4 a-c), visualizing OER activity of β-$Co(OH)_2$ particles on the GC supporting electrode. The micropipet probe (d_{tip} = 120 nm) was equipped with Ag/AgCl QRCEs and filled with 0.1 M KOH. Data extracted from Movie S1 were used to construct Figure 4b in the main text. The data presented are not interpolated.

Movie S2. Spatially-resolved electrochemical (current−voltage) movie (44 × 29 pixels over a 26.4 × 17.4 μm^2 area, hopping distance 600 nm) obtained with the voltammetric (υ = 1 V s^{-1}) hopping mode SECCM configuration (shown in the main text, Figure 4 d-f), visualizing OER activity of β-$Co(OH)_2$ particles on the GC supporting electrode. The micropipet probe (d_{tip} = 440 nm) was equipped with Ag/AgCl QRCEs and filled with 0.1 M KOH. Data extracted from Movie S2 were used to construct Figure 4e in the main text. The data presented are not interpolated.

Section S5. SECCM line scan protocols and additional line scan profiles of particles

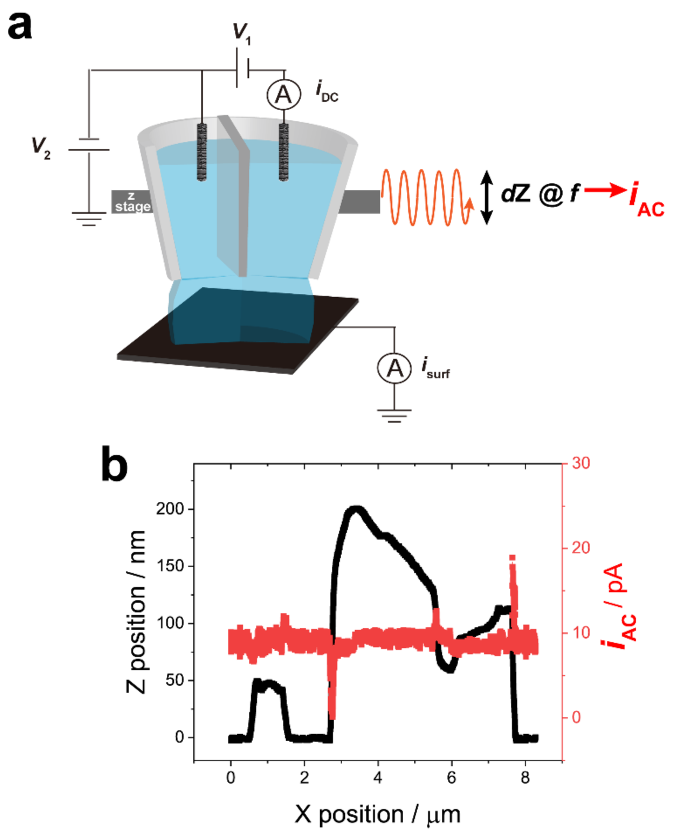

Figure S8 (a) Schematic of SECCM operated in the constant distance scanning mode. During operation, an ionic current (i_{DC}) is induced by applying a voltage bias (V_1) between the two barrels, while the SECCM probe is physically modulated normal to the surface at a fixed amplitude (dZ) and frequency (f), generating an AC current (i_{AC}). i_{AC}, sensitive to the tip-substrate separation, is used as positional feedback to maintain a constant distance during scanning. (b) Z position profiles plotted with concurrently measured i_{AC} during a lateral scan along β-Co(OH)$_2$ particles on the GC surface, demonstrating a stable tip-substrate separation across the particles. Note that (b) was obtained using a probe with a tip diameter (d_{tip}) of 440 nm (V_1 = 50 mV; dZ = 50 nm) filled with 0.1 M KOH, with a lateral translation speed of 20 nm s^{-1}.

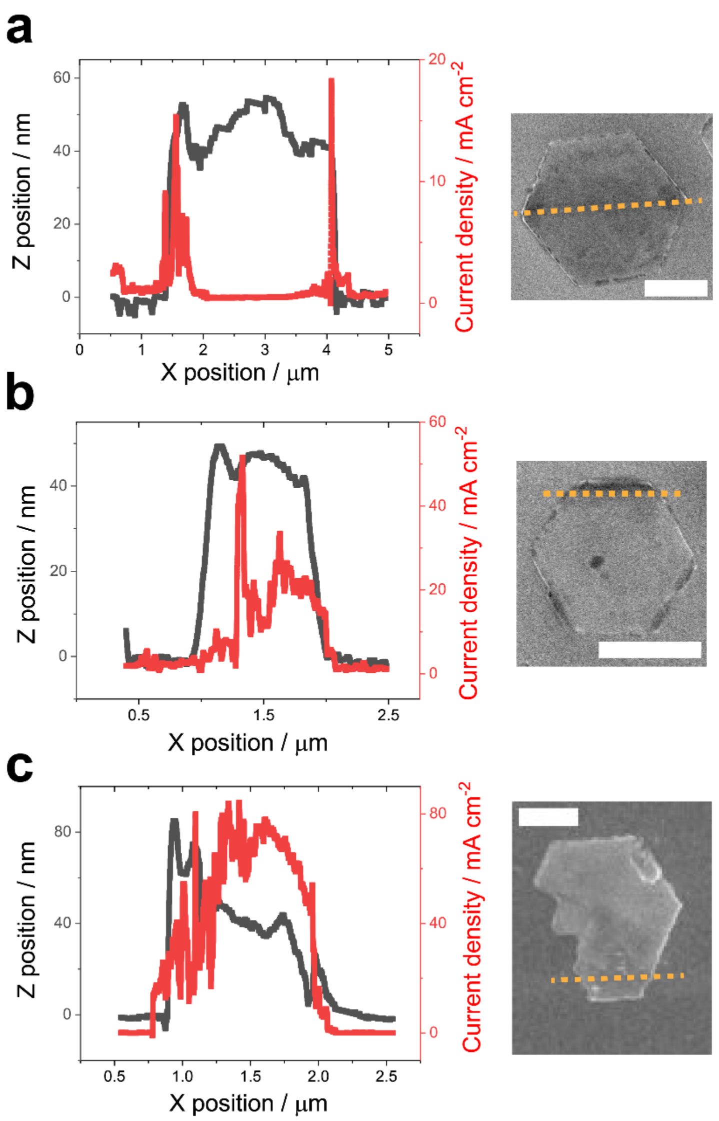

Figure S9. Line scan profiles of current density (solid red trace) and topography (solid black trace) as a function of X-position, taken across (a) a pristine particle, (b) the edge of a pristine particle, and (c) a fragmented particle, along with their corresponding SEM images. Note that the current was normalized to current density based on the SECCM probe size. The SECCM experiments were carried out in the constant distance scanning mode (lateral translation speed = 20 nm s^{-1}) using a dual channel nanopipet probe with d_{tip} = 440 nm, filled with 0.1 M KOH. All scale bars, 1 μm.

References

(1) Gorman, C. B.; Carroll, R. L.; He, Y.; Tian, F.; Fuierer, R. Chemically Well-Defined Lithography Using Self-Assembled Monolayers and Scanning Tunneling Microscopy in Nonpolar Organothiol Solutions. *Langmuir* **2000**, *16*, 6312-6316.

(2) Li, Y.; Morel, A.; Gallant, D.; Mauzeroll, J. Oil-Immersed Scanning Micropipette Contact Method Enabling Long-term Corrosion Mapping. *Anal. Chem.* **2020**, *92*, 12415-12422.

(3) Daviddi, E.; Shkirskiy, V.; Kirkman, P. M.; Robin, M. P.; Bentley, C. L.; Unwin, P. R. Nanoscale electrochemistry in a copper/aqueous/oil three-phase system: surface structure–activity-corrosion potential relationships. *Chem. Sci.* **2021**, *12*, 3055-3069,.

(4) Shkirskiy, V.; Yule, L. C.; Daviddi, E.; Bentley, C. L.; Aarons, J.; West, G.; Unwin, P. R. Nanoscale Scanning Electrochemical Cell Microscopy and Correlative Surface Structural Analysis to Map Anodic and Cathodic Reactions on Polycrystalline Zn in Acid Media. *J. Electrochem. Soc.* **2020**, *167*, 041507.
